# Selection of Reference Genes for Gene Expression Studies related to lung injury in a preterm lamb model

**DOI:** 10.1038/srep26476

**Published:** 2016-05-23

**Authors:** Prue M. Pereira-Fantini, Anushi E. Rajapaksa, Regina Oakley, David G. Tingay

**Affiliations:** 1 Neonatal Research Group, Murdoch Childrens Research Institute, Parkville, Australia; 2 Department of Neonatology, Royal Children’s Hospital, Parkville, Australia; 3 Department of Paediatrics, University of Melbourne, Parkville, Australia

## Abstract

Preterm newborns often require invasive support, however even brief periods of supported ventilation applied inappropriately to the lung can cause injury. Real-time quantitative reverse transcriptase-PCR (qPCR) has been extensively employed in studies of ventilation-induced lung injury with the reference gene 18S ribosomal RNA (18S RNA) most commonly employed as the internal control reference gene. Whilst the results of these studies depend on the stability of the reference gene employed, the use of 18S RNA has not been validated. In this study the expression profile of five candidate reference genes (18S RNA, ACTB, GAPDH, TOP1 and RPS29) in two geographical locations, was evaluated by dedicated algorithms, including geNorm, Normfinder, Bestkeeper and ΔCt method and the overall stability of these candidate genes determined (RefFinder). Secondary studies examined the influence of reference gene choice on the relative expression of two well-validated lung injury markers; EGR1 and IL1B. In the setting of the preterm lamb model of lung injury, RPS29 reference gene expression was influenced by tissue location; however we determined that individual ventilation strategies influence reference gene stability. Whilst 18S RNA is the most commonly employed reference gene in preterm lamb lung studies, our results suggest that GAPDH is a more suitable candidate.

Preterm birth is associated with developmental immaturity of the lung. Functionally this limits the ability of the lung to maintain effective aeration and gas exchange. As a result, preterm newborns often require assisted respiratory support^1^. However, even brief periods of assisted ventilation applied inappropriately to the developmentally and mechanically fragile preterm lung can initiate inflammation and injury^2^,^3^,^4^, via a variety of different mechanisms throughout the lung, which create a gravity-dependent heterogeneous pattern of injury throughout the lung, and influence the benefit of subsequent therapies such as surfactant^5^,^6^. For ventilation-induced lung injury (VILI) to be reduced, the early injurious mechanistic pathways need to be clearly understood firstly in translatable animal models, and ultimately, human infants.

The premature sheep is an established and well-validated model of both respiratory therapies and subsequent lung injury that shares key morphological, mechanical and developmental similarities with preterm infants. Real-time quantitative reverse transcriptase-PCR (qPCR) is a powerful technique essential to increasing our understanding of the interaction between mechanical ventilation and lung injury which has been increasingly used over the past decade^7^,^8^,^9^,^10^,^11^,^12^,^13^,^14^. Whilst qPCR offers many benefits, including high sensitivity, relatively low cost and high time efficiency, qPCR accuracy is influenced by the expression stability of the internal control reference genes. Hence, the identification of suitable reference genes is critical for obtaining reliable results from gene expression studies using qPCR because the expression of reference genes may vary considerably under different experimental conditions, such as different ventilation strategies. 18S ribosomal RNA (18S RNA) has commonly been employed as the reference gene in lamb lung studies^7^,^8^; however its use has not been validated or compared with alternative reference gene candidates.

In this study we perform the first, comprehensive study of reference gene expression under varying ventilation strategies in a preterm lamb model. Evaluation of five reference genes was performed on two geographical and gravity-dependent distinct areas of lung and obtained following a variety of assisted ventilation strategies, by analyzing reference gene stability with three different algorithms (geNorm^15^, NormFinder^16^ and BestKeeper^17^). To demonstrate the influence of reference gene stability on gene normalization, we have examined changes in the relative gene expression of Early growth response protein 1 (EGR1), a commonly employed injury biomarker for VILI, and interleukin 1 beta (IL1B), an early marker of inflammation in preterm lambs and other models of lung injury^1^,^6^,^7^,^18^, when normalized against the most commonly employed reference gene in VILI studies (18S RNA), a consistently high ranked reference gene candidate (Glyceraldehyde 3-phosphate dehydrogenase; GAPDH), and the worst reference gene candidate (Ribosomal Protein S29; RPS29).

## Results

### Selection of candidate reference genes and PCR efficiency

A set of five candidate reference genes of varying functional classes including 18S RNA (18S ribosomal RNA; ribosomal RNA), RPS29 (Ribosomal Protein S29; ribosomal protein), GAPDH (Glyceraldehyde 3-phosphate dehydrogenase; carbohydrate metabolism), ACTB (Beta-Actin; cytoskeletal structural protein) and TOP1 (DNA topoisomerase 1; DNA topoisomerase) were targeted in this study (Table 1). Of these, 18S RNA is the most commonly described reference in preterm lamb models of lung injury^7^,^8^,^9^,^10^,^11^,^12^,^13^,^14^, ACTB and GAPDH had been previously validated in adult sheep lung tissue^19^, RPS29 has been extensively used and validated in small animal models^20^,^21^ and TOP1 studied in a rabbit respiratory model^22^. The correlation coefficient (R^2^) values of all candidates varied from 0.899 to 0.995 across the cDNA diluted points (Table 2). Concurrently PCR efficiency values of all pair-primers varied between 87.1% to 126.5% and amplification varied from 1.871 to 2.316.

### qPCR analysis of reference gene expression in gravity-dependent and independent lung tissue

The expression of five candidate reference genes was assayed in samples of non-dependent and gravity-dependent and lung obtained from unventilated fetal lambs (NI) or lambs exposed to one of five ventilation strategies (V1–V5). Statistical analysis performed on a pool of control and intervention tissue stratified by location is detailed in Table 3. The highest expressing reference gene was 18S RNA with Ct ranges of 12.36 ± 1.14 (non-dependent tissue) and 12.36 ± 0.90 (gravity dependent tissue). RPS29 exhibited the lowest gene expression with detectable Ct ranging between 37.19 ± 1.64 (non-dependent tissue) and 38.97 ± 1.59 (gravity-dependent tissue). GAPDH, ACTB and TOP1 were similarly expressed. Examination of the coefficient of variance (%) suggested that RPS29, GAPDH and TOP1 all exhibited similar variability (4.07–4.90%), whilst gene expression of 18s RNA and ACTB was more variable than the other candidate reference genes (7.11–9.27%).

**Figure 1:**
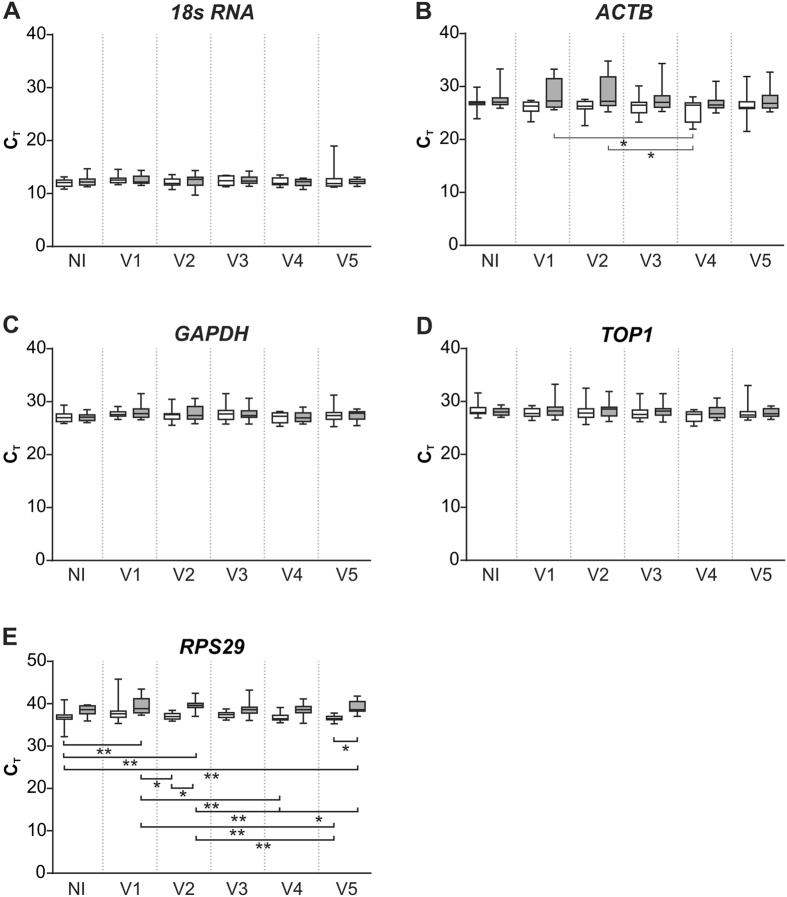
The distribution of 18S RNA (**A**), ACTB (**B**), GAPDH (**C**), TOP1 (**D**) and RPS29 (**E**) reference gene expression levels in non-dependent (white box) and gravity-dependent (grey box) lung tissue obtained from either the NI control group or following a ventilation strategy (ventilation strategies 1–5; V1–V5). The boxes encompass the 25th to 75th percentiles. Whisker caps denote the maximum and minimum values. *P < 0.05, **P < 0.01. N = 10–13/groups.

To determine if sampling location affected candidate reference gene expression, the Ct values for each reference gene was stratified into non-dependent and gravity-dependent groups. The distribution of the Ct values for each of the reference genes is displayed in Fig. 1A–E. Within the NI group there were no influence of sampling location on reference gene Ct distribution. Amongst the ventilation treatment groups, RPS29 gene expression was significantly lower in non-dependent lung tissue when compared with gravity-dependent tissue from the V2 (P = 0.0226) and V5 (P = 0.0102) ventilation groups. Location of tissue sampling did not influence the overall distribution of Ct values of 18S RNA ACT, GAPDH or TOP1.

### Analysis of gene expression stability in non-ventilated tissue samples

Reference gene stability was analyzed using a combination approach that produced an comprehensive ranking based on the geometric mean of results obtained from the comparative delta Ct, GeNorm, NormFinder and BestKeeper analysis programs (Table 4). We began by assessing gene stability of the five candidate reference genes in non-dependent and gravity-dependent tissue samples from the non-ventilated (NI) control group and compared these results with a pooled subset containing qPCR results from both geographical regions. This allowed us to assess if sampling from either non-dependent or gravity-dependent regions influenced reference gene stability. According to the geNorm algorithm, all candidate reference genes, regardless of sampling location meet the programs default limit of <1.5 M = 0.55–1.39). However when considering the results of the BestKeeper algorithm, in which SD_Ct value_ < 1 indicates genes which are stably expressed only GAPDH and 18S RNA were considered stable across both tissue sampling locations. Excluding RPS29, which consistently ranked last, the comprehensive ranking differed between the non-dependent and gravity-dependent tissues. When results from both tissue locations were pooled, the resultant comprehensive ranking did not consistently mirror results from a particular sampling location. GAPDH gene expression was consistently ranked in first or second position across the Delta Ct, GeNorm and NormFinder analyses, and between non-dependent and gravity-dependent tissues and ranked highest in the analysis of the combined tissue set. However, BestKeeper analysis delivered a third level ranking for GAPDH stability across all tissue sets. 18S RNA expression stability was ranked highest by the BestKeeper software due to low standard deviation amongst the Ct values, but exhibited ranking ranging from two to four across the remaining analysis algorithms. The comprehensive gene ranking of most stable to least stable genes in non-ventilated tissue obtained from a gravity non-dependent location was GAPDH > ACTB > TOP1 > 18S RNA > RPS29. The comprehensive ranking in non-ventilated tissues obtained from a gravity-dependent location was TOP1 > GAPDH > 18S RNA > ACTB > RPS29 whilst the comprehensive ranking resulting from a pool containing both geographical locations was GAPDH > 18S RNA > TOP1 > ACTB > RPS29.

### Analysis of gene expression stability within a subset containing both non-ventilated and ventilated tissue samples

To assess the impact of ventilation interventions on reference gene stability we repeated the analysis with a sample group which contained all the NI samples and ventilated lung samples (V1–V5) from either non-dependent or gravity-dependent sample locations and from a pooled group containing both sampling locations (Table 5). We had previously observed that all candidate reference genes in a no intervention situation exhibited stability according to the geNorm algorithm cut-off of M < 1.5. However, following the inclusion of ventilated lung samples to the study, the M values of ACTB and RPS29 were greater than 1.5, indicative of instability. In contrast the results of the BestKeeper analysis performed on NI alone and NI + ventilation study groups were in agreement with ACTB, TOP1 and RPS29 exhibiting instability as determined by SD_Ct value_ > 1. Of note, the inclusion of ventilated tissue with NI samples resulted in alteration to the comprehensive gene ranking in non-dependent tissue such that most stable to least stable gene ranking was 18S RNA > GAPDH > TOP1 > ACTB > RPS29. Similarly in gravity-dependent tissues the ranking altered to be 18S RNA > GAPDH > TOP1 > ACTB > RPS29. These results suggest that ventilation strategies may influence reference gene stability, and that this influence is specific to the tissue location examined.

### Influence of reference gene choice on the reported relative gene expression of early growth factor 1 (EGR1)

#### Choice of reference gene influences the reported EGR1 gene expression in the preterm lamb model

**Figure 2:**
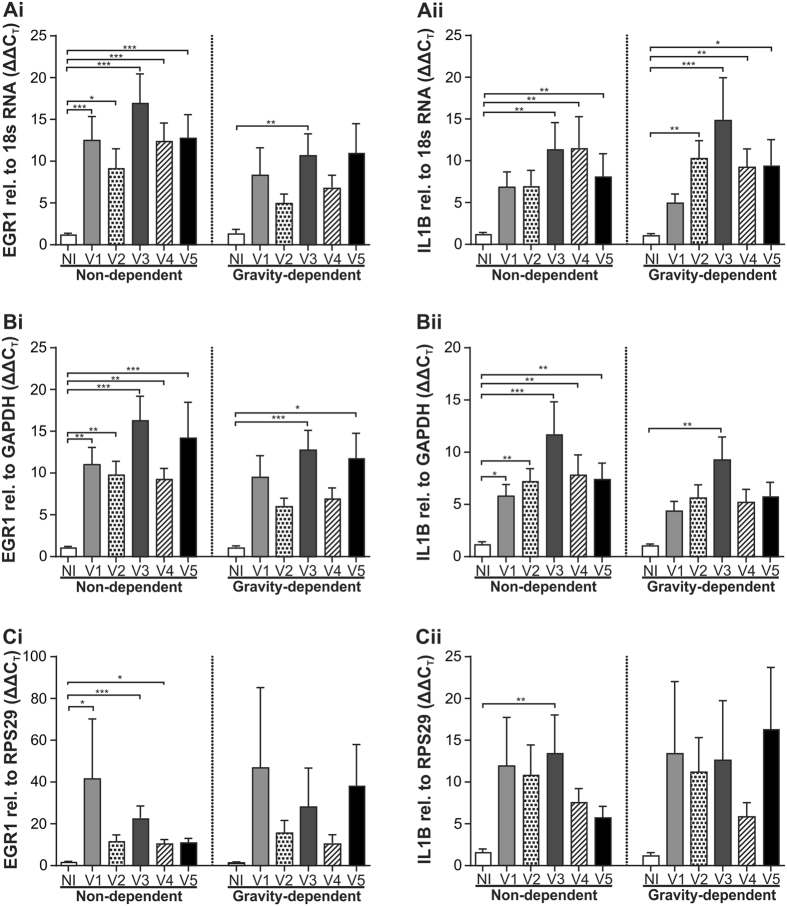
The effect of reference gene choice on reported expression of EGR1 (i) and IL1B (ii) relative to the top ranking candidates 18S RNA (**A**) and GAPDH (**B**) or the lowest ranked candidate RPS29 (**C**) in lung tissue from unventilated control lambs (no intervention; NI) or ventilated lambs (ventilation strategies 1–5; V1–V5). Mean + SEM, N = 10–13/groups. *P < 0.05, **P < 0.01, ***P < 0.001.

To determine if the choice of reference gene used to normalize gene of interest expression significantly alters the statistical outcome reported, we compared normalization of early growth factor 1 (EGR1) and interleukin 1 beta (IL1B) gene expression in unventilated and ventilated tissue against the highest ranked reference gene candidates 18S RNA and GAPDH the worst ranking reference gene candidate, RPS29. The pattern of EGR1 and IL1B gene expression and level of relative fold change was similar when comparing normalization with 18S RNA (Fig. 2Ai,ii) versus normalization with GAPDH (Fig. 2B(i,ii)). However the number of observed statistical differences differed between 18S RNA and GAPDH with a greater number of statistically significant differences in EGR1 gene expression observed in gravity-dependent tissue (two versus one) and similarly increased significance amongst IL1B gene expression in non-dependent tissue samples (five versus three) when normalized to GAPDH rather than 18S RNA. Conversely a greater number of statistically significant differences in IL1B expression were observed when gene expression in gravity dependent tissue was normalized to 18S RNA rather than GAPDH (four versus one). These differences between the top candidates, both of which were the only reference genes determined as stable by both geNorm and BestKeeper, may reflect their differences in PCR efficiency. Not surprisingly, the magnitude of relative fold change and the standard error of the mean were increased when the genes of interest were normalized against the worse candidate, RPS29 (Fig. 2C(i,ii)).

## Discussion

Appropriate reference genes are indispensable for accurate data normalization and thus reliable results in studies of gene expression. In preterm lambs, however, little is known about the ideal genes to use for normalization. The preterm lamb model of lung injury is particularly complex, with reference gene expression potentially influenced by tissue sample location, ventilation strategy, maternal factors and other adjunctive therapies, such as exogenous surfactant and antenatal corticosteroids. Yet, despite these complicating factors, and the increased utilization of qPCR in preterm lamb lung injury analysis, no study to date has provided evidence of reference gene validation in the widely employed pre-term lamb model of ventilation-induced lung injury (VILI).

In initial studies we determined the influence of tissue sample location on the cycle threshold (Ct) of five reference genes which have been commonly employed in lung injury models including the preterm lamb mode (18S RNA), adult sheep model (ACTB and GAPDH), rodent model (RPS29), and rabbit model (TOP1). In the preterm lamb model of lung injury, tissues are commonly sampled from gravity dependent and non-dependent locations; as characteristics specific to the model and human disease, such as gravity-dependent heterogeneity of tidal ventilation, lunch mechanics and aeration may influence the subsequent disease expression. In the current study we report similar median, SD and CV values amongst Ct expression between gravity-dependent and independent lung tissue samples for GAPDH, ACTB, TOP1 and 18S RNA. However, the Ct values for RPS29 differed significantly between non-dependent and gravity-dependent samples in two of the ventilation groups suggesting that reference gene expression can be influenced by tissue location in the lamb model of VILI.

The preterm lamb injury model has been commonly employed in the study of VILI^1^,^6^,^7^,^8^,^9^,^10^,^11^,^14^,^23^,^24^,^25^,^26^,^27^. Application of qPCR in examining lung tissues obtained from the model has the potential to increase our understanding of the molecular alterations which underlie the development of VILI. However, accurate results are dependent on correct reference gene expression. Several studies have reported that stability of commonly used reference genes can significantly vary in given treatments^28^,^29^, however reference gene stability in the setting of VILI has not previously been examined. To assess the influence of intervention on reference gene stability we employed a range of algorithms including geNorm, NormFinder and BestKeeper and compared assessments between a no intervention control cohort and a cohort containing both no intervention controls and ventilated tissue. As each software package implements distinct algorithms, different results can be expected^30^, therefore we employed the geometric mean of the three algorithms together with the comparative delta Ct standard deviation to determine an overall ranking. In both cohorts, ranking results varied between the three algorithms, highlighting the importance of employing more than one software package to identify the most suitable reference gene among a set of candidates. For example, whilst 18S RNA stability in both non-dependent and gravity-dependent tissues from the no intervention group was ranked highest by the BestKeeper software, it was ranked fourth by NormFinder and comparative Delta Ct methods in non-dependent samples and third in gravity-dependent samples, giving it a comprehensive ranking of fourth and third most stable reference gene respectively. 18S RNA has most commonly been employed in qPCR studies of the preterm lamb lung injury model^7^,^8^,^9^,^10^,^11^,^12^,^13^,^14^, however the results from our study of reference gene stability within a cohort of no intervention controls, never exposed to aeration and tidal ventilation, suggests that alternative reference genes would be more suitable in the no intervention (control) setting.

A novel finding of the current study was the influence of intervention on the stability of specific reference genes. In the NI control cohort 18S RNA was the second least stable reference gene, however when ventilated lung samples were included in the analysis, 18S RNA became the most stable candidate. In contrast, GAPDH efficiency calculated using a standard curve technique was less than that of RPS29 (126.5% *versus* 98.7%) however all algorithms tested suggested that GAPDH exhibited consistent stability in both the control cohort and the cohort containing both controls and ventilated tissue, and therefore may be considered to be a better candidate reference gene due to its stability in both the control and experimental setting. As the primary focus of these studies was to explore differences in the stability of commonly employed reference genes in the face of either no intervention or ventilation- we maintained identical reaction conditions across the gene targets and this may have impacted on GAPDH efficiency. To better reflect the experimental situation, efficiency calculations were based on results from representatives of the NI control group and the V3 ventilation group in which ventilation-induced damage is highest. Hence, the discordant results between PCR efficiency and gene stability assessment for GAPDH and RPS29 may reflect the influence of an experimental stress, such as assisted ventilation on PCR parameters. This idea is supported by the observed alterations in 18S RNA gene stability in the face of assisted ventilation, which further suggests that gene stability may be influenced by ventilation intervention in the preterm lamb model of injury. Given the popularity of the preterm lamb as a preclinical model for intervention studies of lung injury this is an important finding and suggests that any new studies investigating alternative interventions should include similar validation of a candidate reference gene panel.

To determine the impact normalization to different reference genes has on the expression pattern of target genes, the relative expression of EGR1 and IL1B was normalized against 18S RNA, GAPDH or RPS29. Overall, the gene expression of EGR1 and IL1B followed a similar pattern when either 18S RNA or GAPDH were employed to normalize gene expression. Interestingly, whilst the statistical significance of EGR1 expression was similar within non-dependent tissue, additional significance was observed when EGR1 was normalized to GAPDH results from gravity-dependent tissue. In contrast, IL1B gene expression exhibited greater significance in non-dependent samples normalized to GAPDH and increased significance in gravity-dependent tissues normalized to 18S RNA. In the current study we employed the ΔΔCt method, Ct values were normalized to both the reference gene and a control group (in this case the no intervention control group). The differences in statistical significance observed may reflect the reduced stability of 18S RNA gene expression in control tissues or alternatively may reflect differences in the PCR efficiency of the two candidate genes. Normalization to RPS29, our least stable reference gene, resulted in large gene expression variation and consequently less statistically significant results, highlighting the usefulness of employing a vigorous analysis of gene stability prior to beginning qRT-PCR studies.

We acknowledge the limitations of the present study. Our choice of reference genes analyzed was limited to five potential candidate genes. Other reference genes, including 60S ribosomal protein (L32)^26^,^31^, or ribosomal protein subunit 15 (RPS15)^25^,^32^,^33^,^34^, have been used in a small number of lamb lung studies and may warrant consideration in the future. Furthermore, due to the developmental plasticity of the model and the geographic tissue response observed within the lung, the results of this study may not be applicable to other ovine or organ models. Certainly expression levels of commonly used reference genes are known to vary across different cell or tissue types, and even within one cell or tissue type when subjected to different conditions^35^. Therefore with the development of new models, and application of interventions, validation studies will need to be repeated.

In conclusion, this study is the first to comprehensively analyze the stability of a panel of reference genes in response to various interventions in dependent and non-dependent lamb lung samples. In the setting of the preterm lamb model of lung injury, reference gene expression of RPS29 was influenced by tissue location. Studies comparing reference gene stability in a control cohort with that of a cohort which also included ventilated lung tissue indicated that ventilation strategies influence reference gene stability. Whilst 18S RNA is the most commonly employed reference gene in preterm lamb lung studies, its stability varied widely between the control cohort and the control/treatment cohort which may influence downstream ΔΔCt calculations. GAPDH displayed similar stability in both cohort studies, suggesting it to be a more suitable candidate for use in the lamb VILI model.

## Methods

### Animal and Sample Collection

The study was performed at the animal research facility of the Murdoch Childrens Research Institute (MCRI; Melbourne, Australia) and approved by the MCRI Animal Ethics committee in accordance with the National Health and Medical Research Committee (Australia) guidelines. Sixty Border-Lecicester/Suffolk 127–129 d lambs (Term ~147 d) were delivered via caesarean section under general anesthesia after standardized fetal instrumentation. Lambs were ventilated supine for 60 minutes from birth with one of five ventilation strategies designed to expose the lambs to varying degrees of lung protection or VILI in early life^6^,^27^. Details of these strategies have been reported previously^27^,^28^,^36^,^37^. In summary, strategies consisted of approaches that either explored the role of applied pressure and time during the first inflation of life (commonly termed a sustained inflation) to clear the fetal lung fluid from the respiratory tree at birth, and aerate the lung (V1–3), or the role of end-expiratory pressure during tidal ventilation to maintain aeration and prevent fluid efflux (V4–5)^6^,^27^. At 60 minutes, lambs were humanely euthanized and tissue samples from standardized gravity-dependent (dorsal) and gravity-independent (ventral) regions of the right lower lobe immediately frozen at −80 °C. Additionally, tissue was sampled from 10 unventilated fetal lambs (no ventilation intervention; NI), euthanized before birth to act as an unaerated control group^24^.

### RNA isolation and cDNA synthesis

All samples were snap-frozen and stored at −80 °C before RNA extraction. RNA was extracted from lamb lung tissue using TRIzol (Invitrogen, Carlsbad, CA, USA). An additional sodium acetate precipitation treatment was incorporated into the TRIzol extraction procedure to ensure complete removal of potential contaminants. RNA concentration was then measured using a NanoDrop ND-1000 Spectrophotometer (Agilent, Santa Clara, CA, USA). Purity of the total RNA was determined by A260/280 and A260/230 ratios and RNA quality confirmed via agarose gel electrophoresis. For each sample, 0.1 μg RNA was reverse-transcribed into complementary DNA (cDNA) with the Transcriptor First Strand cDNA Synthesis Kit (Roche Applied Science, Penzberg, Germany). cDNA was then diluted 1:5 prior to use in the two-step qRT-PCR.

### Screening of candidate reference genes and primer design

Primers were designed using the Roche Universal ProbeLibrary Assay Design Center and the Primer-BLAST tool (http://www.ncbi.nlm.nih.gov/tools/primer-blast/) was employed to confirm the specificity of the primer design.

### Two-step qRT-PCR

Real-time PCR reactions were performed in 384 well plates allowing all samples for one primer set to be evaluated simultaneously. Reactions were carried out in a final volume of 10 μl containing 2.5 μl diluted cDNA, 5 μl FastStart TaqMan Probe Master (Roche Applied Science), 900 nM of each primer and 250 nM of probe mix. The Lightcycler 480 system (Roche Applied Science; regime for all primers, excluding RPS29), comprised of 95 °C for 5 min to activate the polymerase followed by 45 cycles of denaturation at 95 °C for 10 s, annealing at 60 °C for 30 s and extension at 72 °C for 1 s. The cycling program for RPS29 was 95 °C for 5 min to activate the polymerase followed by 50 cycles of denaturation at 95 °C for 10 s, annealing at 57 °C for 60 s and extension at 72 °C for 1 s. In addition, no reverse transcriptase (RT) controls were included in each primer study.

As recommended by the Minimum Information for Publication of Quantitative Real-Time PCR Experiments (MIQE) guidelines^38^ standard curves were generated for each candidate reference gene using the Ct value resultant form duplicate serial dilutions of cDNA obtained from both unventilated and ventilated, gravity-dependent and independent lung tissue (total of n = 6 animals), PCR amplification efficiencies were then determined for all candidate reference genes through the slope of the standard curve with the formula as follows:
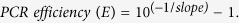


To calculate PCR efficiency and the correlation coefficient (R^2^) and generate the standard curve, Ct values were inputted into Microsoft Excel (Microsoft Office Professional Plus 2013, Redmond, WA, USA).

### Data analysis

Graphpad Prism Software (version 6.0; La Jolla, CA, USA) was employed to determine the mean, standard deviation (SD), co-variance (CV) and to perform one way analysis of variance (ANOVA) testing using raw Ct values from all groups studied and all other statistical analysis unless detailed.

To evaluate the stability of the reference genes samples from non-dependent and gravity-dependent locations from lambs which have subjected to various ventilation strategies, three well-validated assessment logarithms; geNorm^15^, NormFinder^16^ and BestKeeper^17^ each with a unique advantage were employed, together with the comparative ΔCt method. Raw Ct values were used to assess the output of the software packages using the web-based RefFinder platform (http://fulxie.0fees.us/?type=reference). geNorm defines the standard deviation (SD) of the expression ratio of two reference genes as a pair-wise variation under the assumption that the genes tested are not co-regulated and the expression ratio is identical across all samples^39^. The most stably expressed gene yields the lowest M value, and then the two most stable reference genes are determined by step-wise exclusion of the least stable gene^15^. Because of the elimination process, geNorm cannot identify a single suitable reference gene and ends up by suggesting a pair of genes that shows high correlation and should be suitable for normalization of qPCR studies.

NormFinder estimates both intra- and inter-group variations and then combines the two to produce a stability value, which thus represents a practical measure of the systemic error introduced when investigating the gene^16^. Hence, a low stability value reflects low intra- and intergroup variation.

The BestKeeper index is calculated from the geometric mean of the candidates Ct values for each specific sample^39^. The most stable reference genes are the ones with the lowest SD values and highest coefficients of correlation with the BestKeeper index^39^. BestKeeper also uses a statistical algorithm wherein the Pearson correlation coefficient for each candidate reference gene pair is calculated along with the probability of correlation significance of the pair^30^.

To assess the effect of gene reference selection on gene expression patterns, the 2^−ΔΔCt^ method^40^ was used to calculate relative changes in EGR1 gene expression normalized against the candidate reference genes and relative to the no-intervention control group. The resultant data is reported as mean ± standard error of the mean (SEM). Data was compared using a one way analysis of variance (ANOVA). *P* values of <0.05 were considered statistically significant.

## Additional Information

**How to cite this article**: Pereira-Fantini, P. M. *et al.* Selection of Reference Genes for Gene Expression Studies related to lung injury in a preterm lamb model. *Sci. Rep.* **6**, 26476; doi: 10.1038/srep26476 (2016).

## Figures and Tables

**Table 1:** Summary of the reference genes and genes of interest (GOI) investigated in this study.

	Gene	Accession Number	Primer Sequence (5′–3′)	Universal Probe Library Number	Amplicon length (nt)
Reference genes	18S RNA	NR_003286	F-gcaattattccccatgaacg	48	68
			R-gggacttaatcaacgcaagc		
	RPS29	XM_012121306.1	F-gggtaagctgtggcctaaaa	12	73
			R-cggctgcaacgaggtaaa		
	GAPDH	NM_001190390	F-ggcctccaaggagtaaggtc	23	60
			R-tctcttcctctcgtgctcct		
	ACTB	NM_001009784.1	F-gcatggatgatgatattgct	141	109
			R-ccacgatggaagggaagac		
	TOP1	XM_004014835	F-cgagcaggcaacgagaag	12	60
			R-aagcgagcagcaacctacag		
GOI	EGR1	NM_001142506	F-cagcagccccatctactcc	60	60
			R-ggctcagggaagatgtcagt		
	IL1B	NM_001009465	F-gcagtgcggtcatcgtg	44	67
			R-catcacggaagacatgttcg		

The corresponding probe from the Universal Probe Library is also specified (UPL#).

**Table 2:** PCR amplification efficiency of candidate reference gene.

Gene	Slope	R^2^	Efficiency (%)	Amplification
18S RNA	−3.390	0.995	98.7	1.987
RPS29	−4.239	0.899	87.1	1.871
GAPDH	−2.822	0.956	126.5	2.316
ACTB	−3.294	0.994	101.5	2.015
TOP1	−3.413	0.994	98.0	1.968
EGR1	−3.530	0.982	94.4	1.943
IL1B	−4.394	0.895	98.7	1.987

**Table 3:** Candidate reference gene parameters derived from their qPCR.

Gene	Tissue sampling site	Mean Ct	SD	CV (%)
**18S RNA**	Non-dependent	12.36	1.139	9.21
	Gravity-dependent	12.36	0.891	7.21
**RPS29**	Non-dependent	37.19	1.641	4.41
	Gravity-dependent	38.97	1.587	4.07
**GAPDH**	Non-dependent	27.42	1.170	4.27
	Gravity-dependent	27.56	1.177	4.27
**ACTB**	Non-dependent	26.12	1.858	7.11
	Gravity-dependent	27.93	2.590	9.27
**TOP1**	Non-dependent	27.86	1.366	4.90
	Gravity-dependent	28.22	1.363	4.83

**Table 4:** Gene expression stabilities and rankings of reference genes expressed within non-dependent and gravity-dependent lung tissue obtained from the NI group as calculated by the RefFinder tool.

Gene	Comprehensive Ranking		Delta Ct		geNorm		NormFinder		BestKeeper	
	Geomean of Ranking Value	Rank	Average of SD	Rank	M value	Rank	Stability value	Rank	SD	Rank
**GAPDH** (non-dependent)	1.68	1	0.98	2	0.66	1	0.55	2	0.76	3
**GAPDH** (gravity-dependent)	1.68	2	1.11	1	0.55	1	0.27	2	0.63	3
**GAPDH** (All tissues)	1.19	1	1.12	1	0.61	1	0.37	1	0.70	3
**ACTB** (non-dependent)	0.86	2	0.96	1	0.89	3	0.31	1	0.78	2
**ACTB** (gravity-dependent)	4.23	4	1.94	4	1.11	3	1.84	4	1.36	2
**ACTB** (All tissues)	4.00	4	1.57	4	1.06	3	1.29	4	1.05	2
**TOP1** (non-dependent)	2.45	3	1.05	3	0.66	1	0.69	3	1.01	4
**TOP1** (gravity-dependent)	1.00	1	1.09	1	0.55	1	0.27	1	0.60	4
**TOP1** (All tissues)	2.28	3	1.20	3	0.61	1	0.63	3	0.77	4
**18S RNA** (non-dependent)	2.63	4	1.13	4	0.81	2	0.85	4	0.56	1
**18S RNA** (gravity-dependent)	3.00	3	1.14	3	0.63	2	0,32	3	0.80	1
**18S RNA** (All tissues)	1.86	2	1.18	2	0.75	2	0.52	2	0.64	1
**RPS29** (non-dependent)	5.00	5	1.46	5	1.12	4	1.35	5	1.17	5
**RPS29** (gravity-dependent)	4.73	5	1.95	5	1.12	4	1.86	5	0.98	5
**RPS29** (All tissues)	5.00	5	1.89	5	1.39	4	1.73	5	1.39	5

The comprehensive ranking was based on the geometric mean of the comparative delta Ct, GeNorm, NormFinder and BestKeeper results. (N = 10 samples of either non-dependent or gravity-dependent lung tissue/reference gene).

**Table 5:** Gene expression stabilities and rankings of reference genes expressed within non-dependent and gravity-dependent lung tissue obtained from a grouped cohort containing all the ‘no intervention’ (NI) control samples and ventilated lung samples as calculated by the RefFinder tool (N = 70 samples/location and N = 140 samples in the ‘all tissues’ group).

Gene	Comprehensive Ranking		Delta Ct		geNorm		NormFinder		Best Keeper	
	Geomean of Ranking Value	Rank	Average of SD	Rank	M value	Rank	Stability value	Rank	SD	Rank
**GAPDH** (non-dependent)	1.41	2	2.13	1	0.79	1	0.40	1	0.77	3
**GAPDH** (gravity-dependent)	1.41	2	2.83	1	0.74	1	0.37	1	0.90	3
**GAPDH** (all tissues)	1.41	2	2.55	1	0.80	1	0.40	1	0.85	3
**ACTB** (non-dependent)	3.72	4	2.60	4	1.43	3	1.40	2	1.28	2
**ACTB** (gravity-dependent)	3.72	4	3.60	4	1.86	3	2.14	3	2.02	2
**ACTB** (all tissues)	3.72	4	3.25	4	1.74	3	1.92	2	1.58	2
**TOP1** (non-dependent)	3.22	3	2.57	3	1.14	2	1.87	3	0.96	4
**TOP1** (gravity-dependent)	3.22	3	3.55	3	1.44	2	2.48	3	1.19	4
**TOP1** (all tissues)	3.22	3	3.12	3	1.32	2	2.18	3	1.14	4
**18S RNA** (non-dependent)	1.19	1	2.23	2	0.79	1	0.40	1	0.67	1
**18S RNA** (gravity-dependent)	1.19	1	2.90	2	0.74	1	0.37	1	0.66	1
**18S RNA** (all tissues)	1.19	1	2.64	2	0.80	1	0.40	1	0.71	1
**RPS29** (non-dependent)	5.00	5	5.23	5	2.94	4	5.13	4	1.52	5
**RPS29** (gravity-dependent)	5.00	5	7.31	5	4.04	4	7.20	4	2.49	5
**RPS29** (all tissues)	5.00	5	6.35	5	3.58	4	6.23	4	2.18	5

The comprehensive ranking was based on the geometric mean of the comparative delta Ct, GeNorm, NormFinder and BestKeeper results. Gravity dependent and independent lung samples were pooled for the analysis.
